# Healthcare Professionals’ Knowledge, Attitudes, and Practices in the Assessment, and Management of Sickle-Cell Disease: A Meta-Aggregative Review

**DOI:** 10.3390/diseases12070156

**Published:** 2024-07-14

**Authors:** Andrews Adjei Druye, Dorcas Frempomaa Agyare, William Akoto-Buabeng, Jethro Zutah, Frank Odonkor Offei, Bernard Nabe, Godson Obeng Ofori, Amidu Alhassan, Benjamin Kofi Anumel, Godfred Cobbinah, Susanna Aba Abraham, Mustapha Amoadu, John Elvis Hagan

**Affiliations:** 1 Department of Adult Health, School of Nursing and Midwifery, College of Health and Allied Sciences, University of Cape Coast, Cape Coast CC 3321, Ghana; andrews.druye@ucc.edu.gh (A.A.D.); dorcas.agyare@ucc.edu.gh (D.F.A.); fodonkor36@gmail.com (F.O.O.); bnabe@stu.ucc.edu.gh (B.N.); gofori002@stu.ucc.edu.gh (G.O.O.); amidualhassan24@gmail.com (A.A.); 2 Department of Education and Psychology, Faculty of Educational Foundations, College of Education, University of Cape Coast, Cape Coast CC 3321, Ghana; wakotobuabeng@gmail.com (W.A.-B.); jzutah10@gmail.com (J.Z.); 3 Center of Health Research Advancement and Policy, Accra P.O. Box LG949, Ghana; benjamin.anumel@yahoo.com (B.K.A.); sabraham@ucc.edu.gh (S.A.A.); 4 Department of Health, Physical Education and Recreation, University of Cape Coast, Cape Coast CC 3321, Ghana; cobbinahgodfred96@gmail.com (G.C.); amoadu88@gmail.com (M.A.); 5 Department of Public Health, School of Nursing and Midwifery, College of Health and Allied Sciences, University of Cape Coast, Cape Coast CC 3321, Ghana; 6 Neurocognition and Action-Biomechanics-Research Group, Faculty of Psychology and Sports Science, Bielefeld University, Postfach 10 01 31, 33501 Bielefeld, Germany

**Keywords:** attitude, healthcare, knowledge, management, practices, sickle-cell disease

## Abstract

Background: Sickle Cell Disease (SCD) presents significant health challenges globally. Despite its prevalence in diverse geographical regions, there is a paucity of literature synthesizing evidence on healthcare professionals’ knowledge, attitudes, and practices (KAP) toward SCD assessment and management. This meta-aggregative review systematically examined and synthesized existing qualitative research to elucidate healthcare professionals’ KAP regarding SCD assessment and management. Methods: This meta-aggregative review followed Aromataris and Pearson’s guidelines and the PRISMA framework for systematic review reporting. The search was conducted in Scopus, PubMed, Embase, CINAHL Web of Science, Google Scholar, Dimensions AI, and HINARI. Quality appraisal was performed using the Joanna Briggs Institute tool. Results: Healthcare professionals (HCPs) demonstrate varying levels of KAP toward SCD assessment and management. Studies reveal low-to-moderate levels of general knowledge among HCPs, with nurses often exhibiting poorer understanding than physicians. Deficiencies in awareness of specific interventions, such as chemoprophylaxis and prenatal diagnosis, are noted, along with gaps in SCD assessment and diagnosis, particularly in pain management and premarital screening. Attitudes toward SCD patients vary, and practices reveal inconsistencies and deficiencies, including inadequate nutritional counseling and barriers in emergency departments. However, interventions aimed at improving HCPs’ KAP show promise in enhancing understanding and attitudes toward SCD, suggesting potential avenues for improvement. Conclusions: Educational initiatives targeted at both student nurses and practicing healthcare providers, coupled with the implementation of standardized protocols and guidelines, can enhance knowledge acquisition and promote consistent, high-quality care delivery. Future studies should improve the quality of their methods in this area of study.

## 1. Introduction

Sickle Cell Disease (SCD) is a major global health concern due to its significant impact on many affected populations [1]. While SCD was historically associated with malaria-endemic regions in Asia and sub-Saharan Africa, the condition has transcended these origins and is currently found in diverse geographical locations across the world [1,2]. The current global distribution of SCD highlights the need for a comprehensive global approach to address its impact on public health [3].

SCD places affected people at high risk for developing multisystem acute and chronic complications that can lead to significant morbidity and mortality [4]. Therefore, affected people require comprehensive support from healthcare professionals to address challenges [5]. One of the primary reasons for individuals with SCD to actively participate in clinical care is the need for health maintenance and clinical preventive interventions, such as penicillin prophylaxis to prevent infections, and regular screening for early detection of silent organ complications, such as kidney dysfunction and pulmonary hypertension [6,7,8,9].

Additionally, SCD patients require prompt management of acute complications, such as vaso-occlusive crises (VOC) and splenic sequestration as well as comprehensive management of chronic complications, including chronic pain and chronic anemia [10,11,12]. Furthermore, individuals with SCD may experience disability due to complications of the disease, such as avascular necrosis of the joints or stroke-related impairments, which require rehabilitation support from health professionals [13,14].

The complexity of SCD necessitates a multidisciplinary approach to care, involving healthcare professionals from various backgrounds, including physicians, nurses, and allied health professionals [15]. Each member of the healthcare team plays a distinct yet complementary role in providing comprehensive care for patients with SCD [11]. However, effective patient assessment and clinical judgment skills are essential across all disciplines to ensure accurate diagnosis and safe, effective treatment [16,17]. This requires healthcare professionals to possess adequate knowledge and skills specific to the assessment, diagnosis, and management of SCD patients [18].

Healthcare professionals’ knowledge, attitudes, and practices in the assessment and management of SCD are critical determinants of patient well-being and healthcare quality [19]. A healthcare provider’s level of knowledge on SCD pathophysiology, familiarity with evidence-based guidelines, and cultural competence in addressing patients’ unique needs can significantly influence treatment decisions, patient–provider interactions, and overall healthcare experiences [18,20]. Moreover, healthcare professionals’ attitudes toward individuals with SCD, including perceptions of pain management, adherence to treatment, and advocacy for patient rights, can profoundly impact patients’ trust, engagement, and treatment adherence [21].

Evidence has shown that adequate knowledge in the assessment and management of SCD has a direct impact on the attitude and practices of HCPs [22] Thus, health professionals who possess adequate knowledge about SCD and its management, are more likely to implement appropriate practices in their clinical care for improved health outcomes among people with SCD [18]. However, reviews that synthesize evidence from available evidence on the level of knowledge, attitudes, and practices of healthcare providers are scarce. Moreover, there is the need to review interventions that have been directed toward improving the knowledge, attitudes, and practices of healthcare professionals, student nurses, and medics. By synthesizing existing evidence from multiple studies, researchers, and practitioners can discover commonalities and variations in assessment methods and management strategies, as well as areas where improvements are needed to enhance patient care and outcomes. Additionally, findings from a systematic review can inform the development of tailored educational initiatives aimed at addressing knowledge gaps and improving clinical practice among health professionals. Therefore, the aim of this meta-aggregative review is to systematically examine and synthesize existing research studies on health professionals’ knowledge, attitudes, and practices regarding SCD assessment and management.

## 2. Methods

### 2.1. Research Design

This meta-aggregative review was conducted following the guidelines proposed by Munn et al. [23] as advocated by Joanna Briggs Institute (JBI). The guidelines include the following: (1) A clearly defined objective and question, (2) detailed inclusion and exclusion criteria, (3) comprehensive search strategy, (4) quality appraisal of the included studies, (5) analysis of the data extracted, (6) presentation and synthesis of the finding, and (7) transparent reporting of the approach undertaken. The Preferred Reporting Items for Systematic Reviews and Meta-Analyses (PRISMA) guidelines guided the reporting of the search results of this review [24]. The protocol for review was registered in Open Science Framework “https://doi.org/10.17605/OSF.IO/N2JBW (accessed on 5 May 2024)”.

The research question that guided this review: What are the knowledge, attitudes, and practices of assessment and management of people with SCD among healthcare professionals? This research question was defined using the Population, phenomenon of interest, and context framework (PICo). Population: Healthcare professionals; Phenomenon of interest: knowledge, attitudes, and practices on assessment and management of people with sickle cell disease; Context: Global context.

### 2.2. Inclusion and Exclusion Criteria

The inclusion and exclusion criteria were developed to identify studies that addressed this review question. This was informed by the population, concept, and context (PCC) criteria. Table 1 presents the details of the inclusion and exclusion criteria.

### 2.3. Search Strategy

The search for relevant studies was conducted in five main databases, namely, Scopus, PubMed, Embase, CINAHL (Cumulative Index to Nursing and Allied Health Literature), Web of Science. In consultation with a chartered librarian, a search strategy was developed using controlled vocabulary such as Medical Subject Headings (MeSH terms) and keywords identified in a preliminary literature search. Table 2 shows the strategy developed for search in PubMed. A complete search strategy is also presented in Table 2. The search conducted in PubMed was modified for search in other databases. Additional searches were conducted in other Internet-based sources including institutional repositories, HINARI, Dimensions AI, and Google Scholar. Also, the reference lists of the retrieved studies were manually searched to identify relevant literature for inclusion.

The studies that were retrieved were uploaded into the Mendeley referencing software for the removal of duplicates. Following this, titles and abstracts of the remaining studies were screened for inclusion using the eligibility criteria presented in Table 1. This was performed by 16 trained graduate students under the supervision of MA. The graduate students were put into two groups, with eight members in each group. They screened the titles and abstracts independently. This phase of screening was supervised by SAA and MA and reviewed by AAD. The eligibility criteria served as the basis for this screening. The reference lists of the full-text eligible records were checked for additional eligible records. Full-text records of the studies were then screened against the eligibility criteria by two independent groups of authors (BN, JZ, WAB and GOO, AA, FOO). The corresponding authors of the full-text records that were not accessible were contacted for access to the articles. Where misunderstanding occurred, it was resolved by a third reviewer (MA).

### 2.4. Data Extraction

An extraction form developed using Microsoft Word was used to extract the data from the included studies. The form was piloted using five randomly selected articles to assess reliability of the form and the extracted data. Six authors divided into two groups independently performed the data extraction (BN, JZ, WAB and GOO, AA, FOO). Key information that was extracted included the author, year, country, study design, sample size, knowledge, attitudes, and practices of assessment and management of people with sickle cell disease. The extracted data were reviewed by AAD and SAA. Where there were discrepancies, they were resolved by a third reviewer (MA). See Appendix A for details of the extracted data.

Quality appraisal of the included studies was performed after the data extraction using the Quality appraisal tool for qualitative developed by Joanna Briggs Institute (JBI) [25]. This was done to allow the inclusion of studies that were appraised low and medium. This was because those studies contained data that were noteworthy despite the quality of the methods used. Studies were appraised as low, medium, or high. For randomized controlled trials, scores are graded 1–6 (low), 7–8 (moderate), and 9–13 (high). For cross-sectional surveys, scores between 1 and 4, 5 and 6, and 7 and 8 are graded low, moderate, and high, respectively. For non-randomized trials, scores ranging from 1 to 4, 5 to 6, and 7 to 9 represent low, moderate, and high, respectively. All studies that were included were summarized and recorded and concerns about the quality were reported. The quality appraisal was performed independently by two reviewers, and misunderstandings were resolved by discussion. See Appendix B for quality appraisal scores for the included studies.

### 2.5. Data Analysis and Synthesis

Thematic analysis was conducted to identify and analyze patterns within the data extracted from the included studies. This involved systematically coding and categorizing the data to identify recurring themes and patterns related to healthcare professionals’ knowledge, attitudes, and practices regarding SCD assessment and management. The analysis process commenced with familiarization with the data by reading and re-reading the extracted information to gain a comprehensive understanding of the content. Next, initial codes were generated to label and organize meaningful segments of the data related to key concepts such as knowledge, attitudes, and practices. These codes were then collated into potential themes based on their relevance to the research question. Themes were refined through iterative reviewing and discussion among the research team to ensure accuracy and consistency in interpretation. A thematic map or framework was then developed to illustrate the relationships between the identified themes, providing a structured representation of the findings.

A convergent approach to data synthesis for reviews by Hong et al. [26] was followed. Qualitative synthesis was conducted to integrate and interpret the findings from the included studies, focusing on identifying commonalities, differences, and overarching insights across diverse study designs and contexts. This process involved systematically comparing and contrasting the thematic findings to identify convergent and divergent patterns across the data. Through an iterative process of reflection and discussion, the research team synthesized the qualitative data to generate overarching themes and sub-themes that encapsulated the breadth and depth of healthcare professionals’ knowledge, attitudes, and practices concerning SCD assessment and management. Emphasis was placed on capturing the evidence to answer the research question while maintaining transparency and rigor throughout the synthesis process. By consolidating the qualitative synthesis into a coherent narrative, the synthesis aimed to provide a comprehensive understanding of the key issues and implications for healthcare practice and policy.

## 3. Results

### 3.1. Search Results

**Figure 1:**
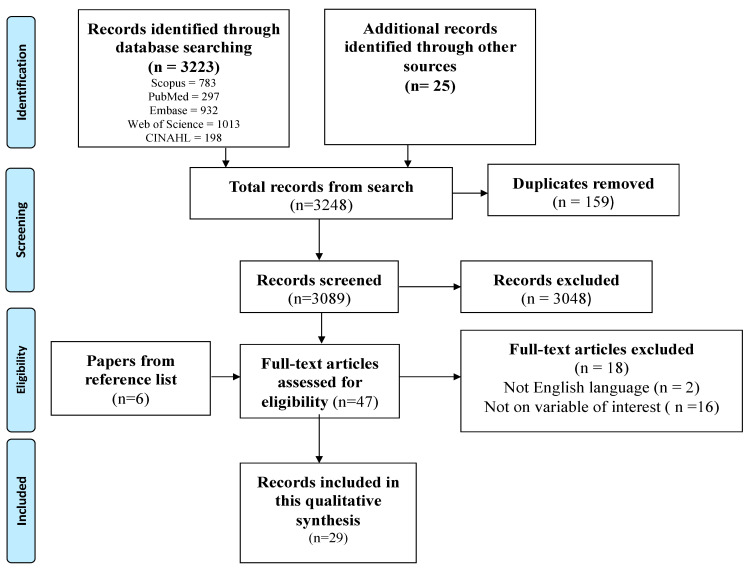
PRISMA flow diagram of search results and screening process.

The search conducted in the five main databases produced 3223 records. An additional 25 records were produced from the search conducted in the additional databases. In all, 3248 records were produced from the search conducted. A total of 159 records were removed using Mendeley Software. Thereafter, 3089 titles and abstracts were screened by the trained graduate students and 3048 records were removed. Thus, 41 full-text records were produced from the screening of titles and abstracts. Checking of reference lists led to the discovery of an additional six eligible records. Finally, 47 full-text records were screened against the eligibility criteria. Eighteen (18) records were excluded, and 29 records were included in this meta-aggregation. Figure 1 presents a flow chart that summarizes the search results and screening process.

### 3.2. Study Characteristics

**Figure 2:**
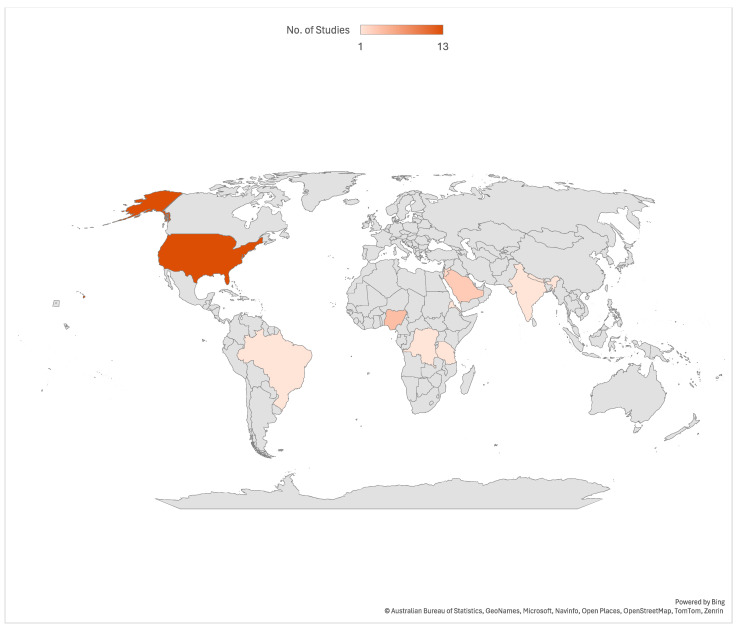
Countries where included studies were conducted.

Most (25) of the included studies are cross-sectional surveys, along with one quasi-experimental design, one randomized post-test-only control group design, one pretest/post-test experimental design, and one single-group pre-test/post-test design. Most (13) of the included studies were conducted in the United States of America. See Figure 2 for more details.

### 3.3. Appraisal Results

Appraisal Scores for Included Studies

**Figure 3:**
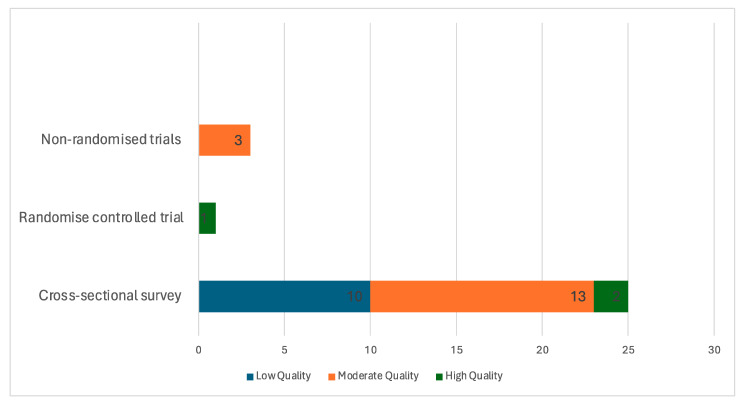
Methodological quality ratings summary of included studies.

Out of the 29 studies included in this review, only 3 studies [27,28,29] received a high methodological appraisal score. Sixteen of the studies received a moderate appraisal score [19,21,22,30,31,32,33,34,35,36,37,38,39,40,41,42], and ten received a low methodological quality score [18,21,43,44,45,46,47,48,49,50]. Most of the included studies lacked methodological strength. See details in Figure 3.

### 3.4. Study Findings

#### 3.4.1. Knowledge of HCPs on SCD Assessment and Management

There is no standardized questionnaire for assessing the KAP regarding SCD assessment and management. The KAP reported in this review are based on questionnaires designed by the included studies to evaluate healthcare providers’ understanding and management of SCD.

##### General Knowledge

Table 3 presents findings on the general knowledge of HCPs regarding SCD assessment and management from five studies [18,19,32,37,49]. Abdeldafie et al. [19] found that only 27.5% of HCPs demonstrated good knowledge of SCD, with a concerning 72.5% of nurses displaying poor knowledge levels. Similarly, Jonathan et al. [18] reported that a mere 25.1% of participants exhibited good knowledge of SCD, while Das et al. [49] found this percentage even lower, at only 4%. In contrast, Stoverock [32] noted that nurses generally possessed a high level of knowledge regarding SCD. Additionally, Isah et al. [37] found that 34.1% of student nurses had good knowledge of SCD, highlighting variations in knowledge levels among different healthcare provider groups.

##### Knowledge of HCPs on SCD Management

Four studies [43,46,47,50] reported on knowledge of HCPs on the management of SCD. Adegoke et al. [43] reported that only 37.9% of HCPs demonstrated good knowledge regarding the nature and care of SCD. Furthermore, Adegoke et al. [43] found that 7.4%, 49.5%, and 67.6% of HCPs were aware of the roles of chemoprophylaxis (folic acid/penicillin), adequate fluids, and malaria prevention, respectively, in SCD care. Additionally, Adegoke et al. [43] noted that only 32.4% and 26.4% of HCPs were knowledgeable about the prenatal and neonatal diagnosis of SCD. In terms of comfort level, Martin et al. [47] found that 54% of providers endorsed a high comfort level in managing vaso-occlusive crises (VOC) associated with SCD. However, Kahsay et al. [46] observed that a significant proportion (57.9%) of nurses had poor knowledge of SCD pain management. Furthermore, Martin et al. [47] revealed that less than 10% of all providers were aware of the recommended timeframe from triage to initial medication administration, indicating gaps in knowledge regarding timely interventions for SCD-related complications.

##### Knowledge of HCPs on SCD Assessment and Diagnosis

Six studies [22,31,33,35,37,47] reported the knowledge of HCPs on SCD assessment and diagnosis. Ngonde et al. [22] reported relatively higher knowledge levels among various categories of HCPs, with physicians, university-level nurses, graduate degree nurses, and high-school-level nurses exhibiting knowledge percentages of 85.7%, 79.3%, 72.8%, and 70.1%, respectively. Among student nurses, Isah et al. [37] found that only 34.3% of student nurses demonstrated good knowledge of premarital screening for SCD. Similarly, Omari [47] found that the majority of student nurses displayed poor knowledge in this domain, indicating a need for targeted educational interventions in pediatric SCD care. Moreover, Martin et al. [33] observed that due to a lack of knowledge, only 25% of respondents appropriately refrained from using vital signs as an indication of a patient’s pain level, suggesting a potential misunderstanding of pain assessment protocols among HCPs. Furthermore, Yaqoob et al. [31] and Shrestha-Ranjit et al. [35] further highlighted deficiencies in the knowledge of nurses regarding pain assessment and management in the context of SCD. Both studies noted poor knowledge levels in this domain.

#### 3.4.2. General Attitude toward SCD Assessment and Management

Abdeldafie et al. [19] found that 56.3% of nurses had fair attitudes, 33.8% had positive attitudes, and 10% had negative attitudes toward sickle-cell patients. Additionally, Das et al. [49] reported that only 46% of healthcare providers held favorable attitudes toward individuals with SCD. Etienne [34] noted that Black individuals were least positive in their attitudes toward SCD, indicating potential cultural factors at play. Furthermore, studies have revealed that nurses reported higher negative attitude scores compared to physicians and exhibited higher levels of negative attitudes toward patients with SCD [21,39,48], including poor attitudes among student nurses toward pediatric assessment and management of SCD. Similarly, Vick et al. [48] also found that nurses exhibited poor attitudes toward patients with SCD.

#### 3.4.3. Attitudes of HCPs on the Management of SCD

Adeyemi et al. [44] found that doctors (21%), nurses (32%), and health workers (32.3%) would accept early termination of affected pregnancies, highlighting differences in acceptance levels among different healthcare professionals. Hazzazi et al. [45] reported that 65.7% of nurses exhibited more negative attitudes toward treating patients with SCD, with emergency providers and internal medicine providers displaying higher concern-raising behaviors. Similarly, However, Razeq et al. [28] reported that most nurses perceived their experience caring for children with SCD as positive.

Kahsay et al. [46] also noted poor attitudes among emergency nurses toward SCD pain management, indicating potential challenges in providing optimal care in emergency settings. Additionally, Pack-Mabien et al. [30] found that the majority of surveyed nurses believed that drug addiction frequently develops in the treatment of SCD pain episodes, but 87% believed it should not be a primary nursing concern, with attitudes influenced by factors such as age, nursing experience, and education level. Glassberg et al. [38] found that most providers self-reported adherence to cornerstones of pain management, such as parenteral opioids and re-dosing opioids, within 30 min if analgesia is inadequate, while adherence was lower for other recommendations. Moreover, Yaqoob et al. [31] observed negative attitudes among nurses toward SCD pain assessment and management. However, Shrestha-Ranjit et al. [35] reported that nurses exhibited a good attitude toward SCD pain assessment and management among children, suggesting positive approaches in specific patient populations.

#### 3.4.4. Attitude of HCPs toward SCD Assessment and Diagnosis

Regarding the assessment and diagnosis of SCD patients, Isah et al. [37] found that 54.4% of respondents exhibited a positive attitude toward premarital screening for SCD. See Table 4 for details.

### 3.5. Practices of HCPs toward SCD Assessment and Management

Seven studies [22,28,30,38,43,46,48] reported on the practice of HCPs on SCD assessment and management. Adegoke et al. [43] highlighted deficiencies in SCD-targeted nutritional counseling and referral practices, with inadequate organization and absence of screening, home visits, and recordkeeping in healthcare centers. Glassberg et al. [38] found that high-volume providers were less likely to re-dose opioids promptly, while pediatric providers showed a higher likelihood of using patient-controlled analgesia (PCA). Kahsay et al. [46] identified barriers in emergency departments, including overcrowding and a lack of pain assessment protocols and tools. Ngonde et al. [22] also reported poor practices across all healthcare providers regarding SCD management. Pack-Mabien et al. [30] highlighted the inadequacy of pain assessment tools as a significant barrier, with 59% of respondents citing this challenge. Meanwhile, Razeq et al. [28] noted that many nurses experienced frustration when caring for children with SCD during painful episodes. Finally, Vick et al. [48] identified shortcomings in the management of SCD-related complications, such as blood transfusion, plasmapheresis, and chelation therapy. See details in Table 5.

### 3.6. Interventions for Improving KAP of HCPs on SCD Management

Several interventions have been implemented to enhance HCPs’ understanding, attitudes, and practices regarding SCD [20,29,36,40,41,42]. In Nigeria, a seminar coupled with free screening significantly boosted student nurses’ knowledge about SCD, with a notable increase from 80.9% to 91.8% post-intervention [42]. Similarly, healthcare providers in Brazil who completed a distance education course showed a 45% increase in SCD knowledge compared to non-completers [36]. In the USA, an educational program aimed at nurses led to an improved understanding of SCD self-management [40]. Additionally, a video intervention in the USA positively impacted clinicians’ attitudes toward adult SCD patients, resulting in decreased negative attitudes and increased positivity [29]. Furthermore, attendance at a two-day SCD conference notably enhanced clinicians’ knowledge and reduced negative attitudes over time, with sustained improvements even two months post-conference [20]. Another effective intervention involved emergency healthcare providers viewing an online video on SCD pain management, which resulted in decreased negative attitudes and increased positive perceptions, sustaining these effects three months after the intervention [41]. See Table 6 for details.

## 4. Discussion

### 4.1. Summary of Findings

The findings suggest that HCPs exhibit variations in KAP toward SCD assessment and management. Regarding general knowledge, studies indicate low-to-moderate levels of understanding among HCPs, with nurses often displaying poorer knowledge compared to physicians. In terms of SCD management, deficiencies are noted in awareness of specific interventions, such as chemoprophylaxis and prenatal diagnosis, highlighting areas for improvement. Similarly, gaps exist in the assessment and diagnosis of SCD, particularly in pain management and premarital screening. Attitudes toward SCD patients vary, with some HCPs showing positive attitudes while others exhibit negative perceptions, especially among nurses. Practices toward SCD assessment and management reveal inconsistencies and deficiencies, including inadequate nutritional counseling and barriers in emergency departments. However, interventions aimed at improving HCPs’ KAP have shown promise in enhancing understanding and attitudes toward SCD, suggesting potential avenues for addressing these challenges.

### 4.2. KAP of HCPs on SCD

The observed variations in KAP among HCPs regarding SCD assessment and management reflect complex dynamics within healthcare systems. The low-to-moderate levels of general knowledge among HCPs, particularly nurses, could be attributed to insufficient training and education on SCD during their professional development [18,19,49]. Nurses, who often provide frontline care, may lack specialized training in SCD management compared to physicians, resulting in poorer knowledge levels [22]. Deficiencies in knowledge of specific interventions, such as chemoprophylaxis and prenatal diagnosis, indicate the need for targeted educational interventions [43]. Chemoprophylaxis, including folic acid and penicillin, plays a crucial role in preventing complications in SCD patients, yet many HCPs lack knowledge of its importance [43]. Similarly, gaps in the assessment and diagnosis of SCD, particularly in pain management and premarital screening, show missed opportunities for early intervention and comprehensive care [31,33].

Attitudes toward patients with SCD significantly impact the quality of care they receive and can influence treatment outcomes. Negative perceptions held by HCPs, particularly nurses, toward SCD patients can stem from various factors, including the perceived complexity of care associated with managing the condition and the emotional toll of caring for chronically ill patients [21]. These negative attitudes may manifest as frustration, bias, or a lack of empathy toward SCD patients, leading to disparities in care delivery and patient dissatisfaction [19]. Nurses, who often play a crucial role in providing direct care to SCD patients, may experience burnout or compassion fatigue due to the chronic nature of the condition and the challenges associated with managing SCD-related complications. As a result, they may inadvertently exhibit negative attitudes toward SCD patients, which can adversely affect patient–provider interactions and undermine the therapeutic relationship [21]. Addressing these attitudinal barriers is essential for promoting patient-centered care and improving health outcomes for individuals living with SCD.

Practices toward the assessment and management of SCD expose the systemic challenges within healthcare systems, revealing a multitude of obstacles that hinder effective care delivery. Studies have highlighted inadequate resources, organizational barriers, and the absence of standardized protocols as key issues impeding optimal SCD management [43,46]. Inadequacies in nutritional counseling and barriers encountered in emergency departments are indicative of broader systemic challenges within healthcare settings, including resource constraints and competing priorities [38,46]. These systemic deficiencies can significantly compromise the quality and continuity of care for individuals living with SCD, leading to suboptimal health outcomes and exacerbating health disparities. The lack of standardized protocols may result in inconsistent approaches to SCD management, contributing to variations in care quality and patient experiences. Addressing these systemic challenges requires multifaceted interventions aimed at improving resource allocation, streamlining care processes, and enhancing healthcare infrastructure to better meet the complex needs of SCD patients. Additionally, fostering interdisciplinary collaboration and promoting patient-centered care models can help overcome organizational barriers and facilitate more comprehensive and holistic approaches to SCD management.

### 4.3. Interventions Aimed at Improving KAP of HCPs

Interventions aimed at improving the KAP of HCPs regarding SCD management have demonstrated significant promise in addressing existing challenges and enhancing patient care outcomes. Educational programs, such as seminars, distance learning courses, and targeted conferences, play a crucial role in enhancing HCPs’ understanding of SCD and promoting positive attitudes toward patients with the condition [21,29,36,41,42]. For instance, the implementation of seminars coupled with free screenings in Nigeria resulted in a substantial increase in student nurses’ knowledge about SCD, highlighting the efficacy of educational initiatives in knowledge enhancement [42]. Similarly, completion of distance education courses in Brazil led to a significant improvement in SCD knowledge among healthcare providers, explaining the relevance of accessible and comprehensive educational resources in promoting a better understanding of the disease [36].

Moreover, standardized protocols and guidelines contribute to improving practices and streamlining care delivery for SCD patients, thereby enhancing overall healthcare quality and patient outcomes [40]. Therefore, the results can be used to advocate for the development and implementation of standardized clinical pathways and protocols that emphasize evidence-based practices in the assessment and management of SCD. Regular screenings, prophylactic treatments, and emergency care protocols that are critical for managing both acute and chronic complications of SCD are needed. By providing clear frameworks for assessment, diagnosis, and management, these protocols help standardize care practices across healthcare settings, reducing variability and ensuring consistency in treatment approaches. Additionally, interventions such as video-based training sessions and online learning programs have been effective in positively impacting clinicians’ attitudes toward SCD patients, leading to decreased negative attitudes and increased positivity among healthcare providers [29,41]. These interventions not only address attitudinal barriers but also foster a more empathetic and patient-centered approach to care, ultimately improving patient–provider interactions and satisfaction.

### 4.4. Recommendation for Policy, Practice, and Education

In terms of practice, addressing deficiencies in SCD management requires the implementation of standardized protocols and guidelines to ensure consistency and quality of care delivery. Healthcare institutions need to prioritize resource allocation and organizational restructuring to overcome systemic barriers that hinder effective SCD management. This includes investing in staff training, updating infrastructure, and promoting interdisciplinary collaboration to facilitate holistic patient care. Additionally, fostering a patient-centered care approach can mitigate attitudinal barriers and improve patient–provider interactions, thereby enhancing treatment outcomes and patient satisfaction. The study revealed significant gaps in general knowledge, awareness of specific interventions, and consistent practices among HCPs. For instance, deficiencies in the understanding of interventions such as chemoprophylaxis and prenatal diagnosis, along with inconsistent practices in pain management and premarital screening, were identified. By addressing these gaps, clinical practice guidelines can be updated to include comprehensive training modules focused on these areas, ensuring that all HCPs, including nurses and physicians, receive the necessary education and resources to improve patient outcomes.

From a policy perspective, there is a need for government intervention to support initiatives aimed at improving SCD care. Policymakers should advocate for the integration of SCD education and training into healthcare curricula, ensuring that all healthcare providers receive comprehensive instruction on SCD management. Furthermore, policies should incentivize the adoption of evidence-based practices and the development of standardized protocols to promote consistency and quality across healthcare settings. Financial support for research into SCD and its management is also essential for advancing knowledge and driving innovation in care delivery. Additionally, policymakers have an opportunity to support continuous professional development for healthcare providers in the field of SCD management. This can be achieved through funding initiatives for ongoing training programs, workshops, and conferences focused on SCD care. By investing in lifelong learning opportunities, policymakers can ensure that healthcare providers stay abreast of the latest advancements in SCD research and treatment modalities. Moreover, policies should encourage collaboration between academic institutions, healthcare organizations, and community stakeholders to foster a multidisciplinary approach to SCD care. By leveraging collective expertise and resources, healthcare systems can develop comprehensive, patient-centered care models that address the complex needs of individuals living with SCD.

In terms of nursing and medical education, there is a critical need to enhance the curriculum to better prepare future healthcare providers for managing SCD. This includes incorporating SCD-specific content into undergraduate and postgraduate education programs, as well as providing continuing education opportunities for practicing nurses and physicians. Simulation-based training and experiential learning activities can help bridge the gap between theory and practice, equipping healthcare providers with the necessary skills and competencies to deliver high-quality care to SCD patients. Additionally, fostering cultural competence and empathy training can help address attitudinal barriers and promote patient-centered care. Further, the study’s synthesis of existing research provides a consolidated source of information on the current state of HCPs’ knowledge and practices regarding SCD. The outcomes can serve as a valuable resource for medical educators and healthcare administrators to design curricula and training programs that are evidence-based and tailored to address the specific deficiencies identified. By improving the baseline knowledge and competencies of HCPs, the overall quality of care for patients with SCD can be enhanced, leading to better health outcomes and patient satisfaction.

### 4.5. Limitations

The review exhibits several limitations that should be considered when interpreting its findings. The restriction to studies published only in English introduces language bias, possibly overlooking valuable literature in other languages. Also, the inclusion of studies with low or moderate methodological quality may introduce biases into the synthesis of findings. Moreover, the inclusion of low- and moderate-rated quality studies necessitates caution when drawing conclusions and making recommendations based on the findings from this review. It is worth noting that the quality appraisal reported in this review may be because of missing statements from the included studies. The predominance of cross-sectional surveys among the included studies, which are sometimes affected by response bias, may further impact the reliability of the findings. In addition, the geographic bias toward studies conducted in the United States may limit the generalizability of the findings to other regions with distinct healthcare systems and cultural contexts. One limitation of this review is the lack of evidence from psychologists and other mental health providers on the assessment of healthcare providers’ knowledge, attitudes, and practices regarding SCD. Given that the studies included in this review originate from different countries, it is important to recognize the potential differences in healthcare systems, study programs, availability of monitoring tools, treatments, care pathways, cost of care, and disease perception. These variations may influence the results and interpretations, underscoring the need to consider both universal and context-specific factors in SCD management. However, a notable strength of the review is its comprehensive search strategy, which involved consultation with a chartered librarian and searches across various databases and sources, enhancing the robustness of evidence synthesis.

### 4.6. Recommendations for Future Studies

Quality studies are needed in order to access the KAP of HCPs on the assessment and management of SCD. Future studies should focus on longitudinal research to track changes in healthcare professionals’ KAP toward SCD assessment and management over time. Additionally, conducting comparative studies across different healthcare settings, utilizing qualitative methods to explore factors influencing attitudes, and evaluating the effectiveness of interventions are crucial. Cross-cultural studies can identify culturally sensitive approaches, while patient-centered research incorporating patient perspectives can inform priorities for improvement. Interdisciplinary collaboration and investigations into long-term outcomes will further enhance understanding and care for individuals with SCD. Future studies could target a broader range of healthcare providers, including mental health professionals, to obtain a more comprehensive understanding of the multidisciplinary approach required for effective SCD management.

## 5. Conclusions

The findings of this review suggest that HCPs demonstrate varying levels of KAP toward sickle cell disease (SCD) assessment and management. Overall, there are low-to-moderate levels of general knowledge among HCPs, with nurses often exhibiting poorer understanding compared to physicians. Deficiencies in awareness of specific interventions, such as chemoprophylaxis and prenatal diagnosis, are evident, highlighting areas for improvement in SCD management. Gaps also exist in the assessment and diagnosis of SCD, particularly in pain management and premarital screening. Attitudes toward SCD patients vary, with some HCPs displaying positive attitudes while others hold negative perceptions, especially among nurses. Practices toward SCD assessment and management reveal inconsistencies and deficiencies, including inadequate nutritional counseling and barriers in emergency departments. However, interventions aimed at improving HCPs’ KAP have demonstrated promise in enhancing understanding and attitudes toward SCD, suggesting potential avenues for addressing these challenges. Educational initiatives targeted at both student nurses and practicing healthcare providers, coupled with the implementation of standardized protocols and guidelines, can enhance knowledge acquisition and promote consistent, high-quality care delivery. Additionally, policy support and interdisciplinary collaboration are essential for overcoming systemic barriers and fostering patient-centered care models. While this review provides valuable insights, its limitations, such as language bias and inclusion of studies with varying methodological quality, underscore the need for cautious interpretation of findings. Moving forward, concerted efforts are needed to address these limitations and implement evidence-based strategies to improve SCD care and ultimately enhance patient outcomes.

## Figures and Tables

**Table 1:** Inclusion and exclusion criteria.

Inclusion Criteria	Exclusion Criteria
Population Studies conducted on: Healthcare professionals All cadres of healthcare professionals Student healthcare professionals	Population Studies conducted on: Only people living with sickle cell disease Only caregivers of people living with sickle cell disease
Concept Assessment and management of people living with sickle cell disease. Knowledge, and/or attitude and/or practice of healthcare professionals in managing people with sickle cell	Concept Management of caregivers of people living with sickle cell
Context Studies in any country in the world Papers published in English Language Papers that have been peer-reviewed and grey literature No time limit All types of study designs	Context Studies that were not published in English Preprint Conference paper Commentaries Letters to editors Reviews

**Table 2:** a: Search strategy for search in PubMed. b: Complete Search strategy conducted in main databases.

(**a**)	
**Search (#)**	**Search Terms**
#1 Search to identify Healthcare professionals	“Healthcare providers” [MeSH Term] OR “Health Personnel” [MeSH Term] OR “Healthcare Personnel” [MeSH Term] OR “Medical Staff” OR “Nurses” OR “Physicians” OR “Health Practitioners” OR “Healthcare Workers” OR “Clinical Staff” OR “Health Service Providers” OR “Healthcare Professionals” OR “Health Professional” OR “Allied Health Professional”.
#2 Search to identify Knowledge, attitude and practices	“Knowledge” [MeSH Term] OR “Awareness” [MeSH] OR “Perception” OR “Beliefs” OR “Behaviors” OR “Practices”
#3 Search to identify Assessment and management of SCD	“Assessment” [MeSH Term] OR evaluation OR diagnosis [MeSH Term] OR “Screening” [MeSH Term] OR “Identification” OR “Management “ [MeSH Term] OR “Treatment” OR care OR intervention” OR “Therapy”
#4 Search to identify Sickle cell disease	“Sickle-cell disease” [MeSH Term] OR “Sickle cell anemia” [MeSH Term] OR “Hemoglobinopathy OR “hemoglobinS disorders”
Overall search	#1 AND #2 AND #3 AND #4 NOT Animal Filter activated: English only
(**b**)	
**Database**	**Search Strategy**
PubMed	((“Healthcare providers” [MeSH Term] OR “Health Personnel” [MeSH Term] OR “Healthcare Personnel” [MeSH Term] OR “Medical Staff” OR “Nurses” OR “Physicians” OR “Health Practitioners” OR “Healthcare Workers” OR “Clinical Staff” OR “Health Service Providers” OR “Healthcare Professionals”) AND (“Knowledge” [MeSH Term] OR “Awareness” [MeSH] OR “Perception” OR “Beliefs” OR “Behaviors” OR “Practices”) AND (“Assessment” [MeSH Term] OR “Evaluation” OR “Diagnosis” [MeSH Term] OR “Screening” [MeSH Term] OR “Identification” OR “Management “ [MeSH Term] OR “Treatment” OR “Care” OR “Intervention” OR “Therapy”) AND (“Sickle-cell disease” [MeSH Term] OR “Sickle cell anemia” [MeSH Term] OR “Hemoglobinopathy” OR “Hemoglobin disorders”)) NOT Animal [Filter activated: English only]
Scopus	TITLE-ABS-KEY((“Healthcare providers” OR “Health Personnel” OR “Healthcare Personnel” OR “Medical Staff” OR “Nurses” OR “Physicians” OR “Health Practitioners” OR “Healthcare Workers” OR “Clinical Staff” OR “Health Service Providers” OR “Healthcare Professionals”) AND (“Knowledge” OR “Awareness” OR “Perception” OR “Beliefs” OR “Behaviors” OR “Practices”) AND (“Assessment” OR “Evaluation” OR “Diagnosis” OR “Screening” OR “Identification” OR “Management” OR “Treatment” OR “Care” OR “Intervention” OR “Therapy”) AND (“Sickle-cell disease” OR “Sickle cell anemia” OR “Hemoglobinopathy” OR “Hemoglobin disorders”) NOT DOCTYPE(ct = “re”) AND LANGUAGE (English))
Embase	(Healthcare providers* OR Health Personnel* OR Healthcare Personnel *OR Medical Staff *OR Nurses* OR Physicians* OR Health Practitioners* OR Healthcare Workers* OR Clinical Staff* OR Health Service Providers* OR Healthcare Professionals*) AND (Knowledge* OR Awareness* OR Perception* OR Beliefs OR Behaviors OR Practices*) AND (Assessment* OR Evaluation* OR Diagnosis* OR Screening* OR Identification* OR Management* OR Treatment* OR Care* OR Intervention* OR Therapy*) AND (Sickle-cell disease* OR Sickle cell anemia* OR Hemoglobinopathy* OR Hemoglobin disorders*) NOT (medline OR animal) AND [embase]/lim AND [english]/lim
CINAHL	(“Healthcare providers” OR “Health Personnel” OR “Healthcare Personnel” OR “Medical Staff” OR “Nurses” OR “Physicians” OR “Health Practitioners” OR “Healthcare Workers” OR “Clinical Staff” OR “Health Service Providers” OR “Healthcare Professionals”) AND (“Knowledge” OR “Awareness” OR “Perception” OR “Beliefs” OR “Behaviors” OR “Practices”) AND (“Assessment” OR “Evaluation” OR “Diagnosis” OR “Screening” OR “Identification” OR “Management” OR “Treatment” OR “Care” OR “Intervention” OR “Therapy”) AND (“Sickle-cell disease” OR “Sickle cell anemia” OR “Hemoglobinopathy” OR “Hemoglobin disorders”) NOT (animal)
Web of Science	TS = (“Healthcare providers” OR “Health Personnel” OR “Healthcare Personnel” OR “Medical Staff” OR “Nurses” OR “Physicians” OR “Health Practitioners” OR “Healthcare Workers” OR “Clinical Staff” OR “Health Service Providers” OR “Healthcare Professionals”) AND TS = (“Knowledge” OR “Awareness” OR “Perception” OR “Beliefs” OR “Behaviors” OR “Practices”) AND TS = (“Assessment” OR “Evaluation” OR “Diagnosis” OR “Screening” OR “Identification” OR “Management” OR “Treatment” OR “Care” OR “Intervention” OR “Therapy”) AND TS = (“Sickle-cell disease” OR “Sickle cell anemia” OR “Hemoglobinopathy” OR “Hemoglobin disorders”) NOT (ANIMAL)

**Table 3:** Knowledge of HCPs on SCD assessment and management.

Theme	Findings	Authors
General knowledge	27.5% had good knowledge of SCD.	[19]
	72.5% of nurses had poor knowledge score levels.	[19]
	Only 25.1% had good knowledge of SCD.	[18]
	Only 4% had good knowledge of SCD.	[49]
	Nurses had high knowledge of SCD disease.	[32]
	34.1% of student nurses have good knowledge of SCD.	[37]
Knowledge of SCD Management	37.9% had good knowledge of the nature and care of the disease.	[43]
	7.4%, 49.5%, and 67.6% knew about the role of chemoprophylaxis (folic acid/penicillin), adequate fluids, and malaria prevention, respectively, in SCD care.	[43]
	32.4% and 26.4% knew that SCD can be diagnosed in the prenatal and neonatal periods, respectively.	[43]
	54% of providers endorsed a high comfort level in managing VOC.	[33]
	Majority of student nurses had adequate knowledge about the home management of SVOC among people with SCD.	[50]
	Less than 10% of all providers knew the recommended timeframe from triage to initial medication administration.	[33]
	57.9% of the nurses had poor knowledge of SCD pain management.	[46]
Knowledge of SCD assessment and diagnosis	34.3% of student nurses had good knowledge of premarital screening for SCD.	[37]
	All the HCPs: 85.7%, 79.3%, 72.8%, and 70.1% for physicians, university-level nurses, graduate degree nurses, and high-school-level nurses, respectively.	[22]
	Student nurses had poor knowledge of pediatric assessment and management.	[47]
	Only 25% of respondents appropriately did not use vital signs as an indication of a patient’s pain level.	[33]
	Nurses had poor knowledge of SCD pain assessment and management.	[31]
	Nurses had insufficient knowledge of pain assessment and management of SCD among children.	[35]

Attitudes of HCPs on the Assessment and Management of SCD.

**Table 4:** Attitudes toward assessment and management of patients with SCD.

Theme	Findings	Authors
General attitudes of HCPs toward SCD	56.3%, 33.8%, and 10 of nurses had fair, positive, and negative attitudes toward sickle-cell patients.	[19]
	Only 46% had favorable attitude toward people with SCD.	[49]
	Blacks were least positive in SCD attitude.	[34]
	Nurses had higher negative attitude scores than physicians.	[39]
	Nurses had high levels of negative attitudes toward patients with SCDs.	[21]
	Student nurses had poor attitudes toward pediatric assessment and management.	[47]
	Nurses had poor attitude toward patients with SCD.	[48]
Attitude toward the management of SCD	21% of doctors would accept early termination of affected pregnancy, and 32% and 32.3% of nurses and health workers would accept termination of affected pregnancy, respectively.	[44]
	65.7% of the nurses had more negative attitudes toward treating patients with SCD. Emergency providers and internal medicine providers had higher concern-raising behaviors.	[45]
	Emergency nurses had poor attitude toward SCD pain management.	[46]
	The majority (63%) of the surveyed nurses believed that drug addiction frequently develops in the treatment of sickle cell pain episodes. 87% of the respondents believed drug addiction should not be a primary nursing concern when caring for a patient with sickle cell pain episodes. The belief that drug addiction should be a primary nursing concern in the management of sickle cell pain episodes was influenced by age, years of active nursing experience, and education.	[30]
	Most nurses (77%) perceived their experience caring for children with SCD as positive.	[28]
Attitude toward pain management of SCD	Most providers self-reported adherence to the cornerstones of SCD pain management including parenteral opioids (90%) and re-dosing opioids within 30 min if analgesia is inadequate (85%). Self-reported adherence was lower for other recommendations including use of patient-controlled analgesia (PCA), acetaminophen, NSAIDs, and hypotonic fluids when euvolemic.	[38]
	Nurses had negative attitudes toward SCD pain assessment and management.	[31]
	Nurses have good attitude toward SCD pain assessment and management among children.	[35]
Attitude toward diagnosis and assessment of SCD patients.	54.4% of respondents had good attitude regarding premarital screening for SCD.	[37]

**Table 5:** Practices of HCPs toward assessment and management of SCD.

Practices	Authors
SCD-targeted nutritional counseling and referral to secondary/tertiary hospitals were poor and unorganized. No center offered SCD screening, home visits, or recordkeeping.	[43]
High-volume providers (those who see more than one SCD patient per week) were less likely to re-dose opioids within 30 min for inadequate analgesia. Pediatric providers were 6.6 times more likely to use PCA for analgesia.	[38]
Perceived barriers to adequate pain management in emergency department were overcrowding, lack of protocols for pain assessment, high nursing workload, and lack of pain assessment tools.	[46]
All the participants showed poor practices on SCD.	[22]
59% of the respondents reported that an inadequate pain assessment tool was the greatest barrier in the management of sickle cell pain episodes.	[30]
Many nurses (65%) felt frustrated about caring for these children during painful episodes.	[28]
Poor management of blood transfusion, plasmapheresis, and chelation therapy.	[48]

**Table 6:** Interventions aimed at improving SCD KAP among HCPs.

Authors	Intervention	Results
[42] Nigeria (Student Nurses)	To assess the effect of health education and provision of free sickle cell hemoglobin screening on knowledge of sickle cell disorder, and attitude toward sickle cell hemoglobin screening (Seminar and free screening).	80.9% and 91.8% knowledge at baseline and post-intervention, respectively.
[36] Brazil (Healthcare providers)	Assess the impact of a distance education course.	SCD professional healthcare providers who concluded the distance course had a significantly higher SCD knowledge score (45%) when compared to those who did not successfully conclude the course.
[40] USA (Nurses)	To create an educational program intended to educate nurses to improve their knowledge regarding the self-management of SCD.	Nurses had improved knowledge about the self-management of SCD after the education program.
[29] USA (nurses and house staff)	To assess the impact of video intervention to improve clinician attitudes toward adult SCD patients. An 8 min video depicting a clinician expert and patients discussing challenges in seeking treatment for sickle cell pain.	Compared to the control group, the intervention group exhibited decreased negative attitudes, decreased endorsement of certain patient behaviors as “concern-raising”, and increased positive attitudes toward sickle cell patients.
[20] Nurses in ICU surgical unit	Compare clinicians’ SCD knowledge and attitudes toward patients with SCD, before attending a two-day conference on SCD (T1), to immediately post-conference (T2), and 2 months post-conference (T3).	Overall, knowledge scores were significantly improved as well as significantly increased between T1 and T2 and T1 and T3. Negative attitudes trended lower over the three time points, but a significant decrease in the negative attitudes score was only noted between T1 and T3. Attendance at an educational SCD conference was an effective means to improve knowledge and decrease negative attitudes among clinicians. These differences were maintained at 2 months post-conference.
[41] USA (Emergency HCPs)	To measure pre-intervention and post-intervention providers’ attitudes toward patients with sickle pain crises. ED providers viewed an eight-minute online video that illustrated challenges in sickle cell pain management, perspectives of patients and providers, as well as misconceptions and stereotypes of which to be wary.	Negative attitude scoring decreased from baseline, positive attitudes improved, and endorsement of red-flag behaviors decreased. Results were statistically significant and sustained on repeat testing three months after intervention.

## Data Availability

All references to data have been included in the study.

## Appendix A

**Table A1:** Extracted data from included studies.

Authors, Year of Publication, and Country	Purpose of the Study	Design	Population	Sample Size	Knowledge	Attitude	Practice
Abdeldafie et al. [19] Saudi Arabia	To determine the knowledge of nurses and their attitudes toward SCD patients	Cross-sectional survey	Nurses	240	72.5% of nurses had poor knowledge score levels and 27.5% had good knowledge.	56.3%, 33.8%, and 10 of nurses had fair, positive, and negative attitudes toward sickle cell patients.	
Abiola et al. [42] Nigeria	To assess effect of health education and provision of free sickle cell hemoglobin screening on knowledge of sickle cell disorder, and attitude toward sickle cell hemoglobin screening (Seminar and free screening).	Quasi-experimental design	Student nurses	104	80.9% and 91.8% knowledge at baseline and post-intervention, respectively.		
Adegoke et al. [43] Nigeria	To assess knowledge of primary healthcare providers on sickle cell disease.	Cross-sectional survey	Nurses/midwives, community health officers, community extension workers, dental technicians, laboratory attendants, health records officers and community health assistants	182	37.9% had good knowledge of the nature and care of the disease. Only 32.4% and 26.4% knew that SCD can be diagnosed in the prenatal and neonatal periods, respectively. Also, 37.4%, 49.5%, and 67.6% knew about the role of chemoprophylaxis (folic acid/penicillin), adequate fluids, and malaria prevention, respectively, in SCD care.		SCD-targeted nutritional counseling and referral to secondary/tertiary hospitals were poor and unorganized. No center offered SCD screening, home visits, or recordkeeping.
Adeyemi et al. [44] Nigeria	To assess the knowledge and attitude of female health workers toward prenatal diagnosis of SCD.	Cross-sectional survey	Doctors, nurses, and other healthcare workers	276		21% of doctors would accept early termination of affected pregnancy, and 32% and 32.3% of nurses and health workers would accept termination of affected pregnancy, respectively.	
Boyd [27] USA	To explore nurses’ attitudes and knowledge of pain in SCD.	Cross-sectional survey	Nurses	79	No significant differences in positive or negative attitudes were identified between those who scored lower and those who scored higher on the SCD knowledge test.		
Das et al. [49] India	To assess registered nurses’ knowledge and attitude regarding the care of sickle cell disease.	Cross-sectional survey	Nurses	100	Only 4% had good knowledge of SCD.	Only 46% have favorable attitude toward people with SCD.	
Diniz et al. [36] Brazil	To evaluate the impact of the distance education course “SCD: Primary Health Care Line” on knowledge acquisition by professional healthcare providers.	Cross-sectional survey	Physicians, nurses, dentists, social assistants, psychologists, physical therapists, physical educators, dieticians and others	300	Professional healthcare providers who concluded the distance course had a significantly higher DFConhecimento score (45%) when compared to those who did not successfully conclude the course.		
Etienne [34] USA	To assess nurses’ knowledge and attitude toward people with SCD.	Cross-sectional survey	Nurses	109	About 64.3 percent of the participants were knowledgeable about SCD. Knowledge of SCD was not shown to significantly correlate with attitudes related to caring for the SCD patient.	Blacks were least positive in SCD attitude.	
Freiermuth et al. [39] USA	To validate a survey that measures attitudes toward patients with SCD among ED providers.	Cross-sectional survey	Nurses and physicians	215		Nurses had higher negative attitude scores than physicians.	
Glassberg et al. [38] USA	To assess provider attitudes and self-reported analgesic practices toward patients with SCD.	Cross-sectional survey	Emergency providers	722		Most providers self-reported adherence to the cornerstones of SCD pain management including parenteral opioids (90%) and re-dosing opioids within 30 min if analgesia is inadequate (85%). Self-reported adherence was lower for other recommendations including use of patient-controlled analgesia (PCA), acetaminophen, NSAIDs, and hypotonic fluids when euvolemic.	Emergency providers in the highest quartile of negative attitudes were 20% less likely to re-dose opioids within 30 min for inadequate analgesia. High-volume providers (those who see more than one SCD patient per week), were less likely to re-dose opioids within 30 min for inadequate analgesia. Pediatric providers were 6.6 times more likely to use PCA for analgesia.
Hamid et al. [50] Saudi Arabia	To assess the knowledge of nursing students about home management and prevention of vasoocclusive crisis of sickle cell disease.	Cross-sectional survey	Student nurses	167	The nursing students had adequate knowledge about the home management and prevention of sickle cell disease vaso-occlusive crises.		
Haywood et al. [29] USA	To assess the impact of video intervention to improve clinician attitudes toward adult SCD patients. An 8 min video depicting a clinician expert and patients discussing challenges in seeking treatment for sickle cell pain.	Randomized post-test only control group design.	Nurses and house staff	276		Compared to the control group, the intervention group exhibited decreased negative attitudes; decreased endorsement of certain patient behaviors as “concern-raising”; increased positive attitudes toward sickle cell patients.	
Hazzazi et al. [45] Saudi Arabia	To explore physicians’ and nurses’ perceptions and attitudes toward sickle cell patients.	Cross-sectional survey	Nurses and physicians	244		65.7% of the nurses had more negative attitudes. Those treating primarily. children had higher positive attitudes than those treating adults or treating both. Emergency providers and internal medicine providers had higher concern-raising behaviors.	
Isah et al. [37] Nigeria	To explore student nurses’ knowledge and attitudes regarding Premarital Screening for SCD.	Cross-sectional survey	Student nurses	176	Only about one-third (34.1%) of respondents had good knowledge of SCD, and 34.3% of respondents had good knowledge of premarital screening for SCD.	54.4% of respondents had good attitude regarding premarital screening for SCD.	
Jenerette et al. [21] USA	To determine if there are significant differences in nurse attitudes toward patients with SCD.	Cross-sectional survey	Nurses at ICU and surgical units	77		Nurses have high levels of negative attitudes toward patients with SCDs.	
Jenerette et al. [20] USA	To compare clinicians’ SCD knowledge and attitudes toward patients with SCD, before attending a two-day conference on SCD (T1), to immediately post- conference (T2), and 2 months post-conference (T3).	Cross-sectional survey	Nurses	59		Overall, knowledge scores were significantly improved as well as significantly increased between T1 and T2 and T1 and T3 (*p* = 0.01). Negative attitudes trended lower over the three time points, but a significant decrease in the negative attitudes score was only noted between T1 and T3. attendance at an educational SCD conference was an effective means to improve knowledge and decrease negative attitudes among clinicians. These differences were maintained at 2 months post-conference.	
Jonathan et al. [18] Tanzania	To assess healthcare workers’ knowledge and resource availability for care of SCD at health facilities.	Cross-sectional survey	Nurses and clinicians	490	Only 25.1% had good knowledge of SCD. The odds of good knowledge were lower among nurses, and diploma holders, and higher in those with 5–9 years’ experience.		
Kahsay and Pitkäjärvi [46] Eritrea	To assess the emergency nurses’ knowledge, attitude, and perceived barriers regarding pain management.	Cross-sectional survey	Emergency nurses	126	A mean score of 80% or higher was not achieved by any of the participants. 57.9% of the nurses received a score of less than 50%. Knowledge level of the emergency nurses was poor in SCD pain management. Nurses who had previous training regarding pain scored significantly higher knowledge levels compared to those without training.	Emergency Nurses had poor attitude toward SCD pain management. Nurses with Bachelor’s Degree had significantly higher knowledge and attitude level compared to the nurses at the Diploma and Certificate level.	Perceived barriers to adequate pain management in emergency department were overcrowding, lack of protocols for pain assessment, high nursing workload, and lack of pain assessment tools.
Martin et al. [33] USA	To assess healthcare provider awareness about SCD pain management.	Cross-sectional survey	Emergency nurses, resident trainees, and staff attendings.	52	54% of providers endorsed a high comfort level in managing VOC, with staff and nurses more likely to report this than trainees. Less than 10% of all providers knew the recommended timeframe from triage to initial medication administration. Only one-fourth of all respondents appropriately did not use vital signs as an indication of a patient’s pain level.		
McCullough et al. [40] USA	To create an educational program intended to educate nurses to improve their knowledge regarding the self-management of SCD by patients.	Pre-test/post-test experimental design	Nurses	19	Nurses had improved knowledge about the self-management of SCD after the education program.		
Ngonde et al. [22] Democratic Republic of Congo	To assess the levels of knowledge and practices of SCD and to identify determinants of the practices among primary HCPs.	Cross-sectional survey	Nurses and physicians	318	Physicians had an average level of knowledge about the epidemiology of SCD (65.8%). Nurses with a university’s degree (60.0%) and a graduate degree (59.6%) had an average level of knowledge about epidemiology. All the HCPs showed a high level of knowledge of the clinical manifestations of SCD: 85.7%, 79.3%, 72.8%, and 70.1% for physicians, university level nurses, graduate degree nurses, and high school level nurses, respectively. The proportion of high-school- and graduate-level nurses with an average level of knowledge about the diagnosis, and management of SCD was 52.8% and 55.9%, respectively.		All the participants showed poor practices on SCD. Knowledge of SCD as a significant predictor of better practice for physicians. Knowledge of SCD and duration of work experience were significant predictors of better practices among nurses.
Al-Omari [47] Jordan	To test the nursing students’ knowledge and attitudes toward children’s pain management.	Cross-sectional survey	Student nurses	101	Student nurses have poor knowledge of pediatric assessment and management.	Student nurses have poor knowledge of pediatric assessment and management.	
Pack-Mabien et al. [30] USA	To determine whether nurses’ attitudes influence their practice when caring for patients with sickle cell pain episodes.	Cross-sectional survey	Student nurses, registered nurses, licensed practical nurses, and adult pediatric nurses	77		The majority (63%) of the surveyed nurses believed that drug addiction frequently develops in the treatment of sickle cell pain episodes. 87% of the respondents believed drug addiction should not be a primary nursing concern when caring for a patient with sickle cell pain episodes. The belief that drug addiction should be a primary nursing concern in the management of sickle cell pain episodes was influenced by age, years of active nursing experience, and education.	59% of the respondents reported that an inadequate pain assessment tool was the greatest barrier in the management of sickle cell pain episodes.
Razeq et al. [28] Jordan	To examine nurses’ attitudes toward caring for children with sickle cell disease (SCD) and SCD pain management in those with vaso-occlusive pain.	Cross-sectional survey	Nurses	298		Most nurses (77%) perceived their experience caring for children with SCD as positive.	Many nurses (65%) felt frustrated about caring for these children during painful episodes. Participants identified workload and inadequate time as limiting their ability to address the analgesic needs of children with SCD. Receiving structured education specialized in pain management and more years of experience in nursing significantly predicted less hesitancy in administering opioid-based analgesia.
Shrestha-Ranjit et al. [35] Nepal	To explore knowledge and attitudes regarding pediatric pain assessment and management among nurses at a tertiary children’s hospital	Cross-sectional survey	Nurses	140	Nurses had insufficient knowledge and attitudes that did not reflect best practice regarding pain assessment and management in children		
Singh et al. [41] USA	To measure preintervention and post-intervention providers’ attitudes toward patients with sickle pain crises. ED providers viewed an eight-minute online video that illustrated challenges in sickle cell pain management, perspectives of patients and providers, as well as misconceptions and stereotypes of which to be wary.	Single-group pretest/post-test design	Emergency department HCP	96		Negative attitude scoring decreased from baseline. Positive attitudes improved. Endorsement of red-flag behaviors decreased. Results were statistically significant and sustained on repeat testing three months after intervention.	
Stoverock [32] USA	To examine nurses’ knowledge of SCD.	Cross-sectional survey	Nurses	31	Participants had high knowledge of SCD disease.		
Vick et al. [48] USA	To understand the perceived NP competencies and attitudes toward patients living with SCD.	Cross-sectional survey	Nurses	32		Nurses have poor attitude toward patients with SCD.	Management: blood transfusion, plasmapheresis, and chelation therapy.
Yaqoob and Nasaif [31] Kingdom of Bahrain	To assess the level of knowledge and attitudes of nursing staff regarding pain assessment and management of patients with SCD during sickling crisis.	Cross-sectional survey	Nurse	30	Nurses had poor knowledge of SCD pain assessment and management.	Nurses had negative attitudes toward SCD pain assessment and management.	

## Appendix B

**Table A2:** Appraisal of cross-sectional studies.

Author(s)	Q1	Q2	Q3	Q4	Q5	Q6	Q7	Q8	Total Score	Decision to Include (Yes/No)
Boyd [27]	Yes	Yes	Yes	Yes	Yes	Yes	Yes	Yes	High	Include
Razeq et al. [28]	Yes	Yes	Yes	Yes	Yes	Yes	Yes	Yes	High	Include
Adegoke et al. [43]	Yes	Yes	No	No	No	No	No	Yes	Low	Include
Adayemi et al. [44]	Yes	Yes	No	No	No	No	No	Yes	Low	Include
Hazzazi et al. [45]	Yes	Yes	Yes	Yes	Yes	Yes	Yes	Yes	Low	Include
Jenerrete et al. [20]	Yes	Yes	Yes	Yes	No	No	Yes	Yes	Low	Include
Kahsay and Pitkäjärvi [46]	Yes	Yes	Yes	Yes	No	No	Yes	Yes	Low	Include
Al-Omari [47]	Yes	Yes	Yes	Yes	No	No	Yes	Yes	Low	Include
Vick et al. [48]	No	No	Yes	Yes	No	No	Yes	Yes	Low	Include
Jonathan et al. [18]	Yes	Yes	Yes	No	Yes	Yes	Yes	Yes	Low	Include
Das et al. [49]	Yes	Yes	No	Yes	No	No	No	Yes	Low	Include
Hamid et al. [50]	Yes	Yes	No	Yes	No	No	No	Yes	Low	Include
Abdeldafie and Alaajmi [19]	Yes	Yes	Yes	No	No	No	Yes	Yes	Moderate	Include
Pack-Mabien et al. [30]	Yes	Yes	No	Yes	No	No	No	Yes	Moderate	Include
Yaqoob and Nasaif. [31]	Yes	Yes	Yes	Yes	No	No	Yes	Yes	Moderate	Include
Stoverock et al. [32]	Yes	Yes	Yes	Yes	No	No	Yes	Yes	Moderate	Include
Martin et al. [33]	Yes	Yes	Yes	Yes	No	No	Yes	Yes	Moderate	include
Ngonde et al. [22]	Yes	Yes	Yes	Yes	No	No	Yes	Yes	Moderate	include
Etienne [34]	Yes	Yes	Yes	Yes	No	No	Yes	Yes	Moderate	Include
Shrestha-Ranjit et al. [35]	Yes	Yes	Yes	Yes	No	No	Yes	Yes	Moderate	Include
Diniz [36]	Yes	Yes	Yes	Yes	No	No	Yes	Yes	Moderate	Include
Isah et al. [37]	Yes	Yes	Yes	No	No	No	Yes	Yes	Moderate	Include
Glassberg et al. [38]	Yes	Yes	Yes	Yes	No	No	Yes	Yes	Moderate	Include
Freiermuth et al. [39]	Yes	Yes	Yes	Yes	No	No	Yes	Yes	Moderate	Include
Jenerette et al. [21]	Yes	Yes	Yes	Yes	No	No	Yes	Yes	Moderate	Include

**Table A3:** Appraisal of non-randomized controlled trial (JBI).

Author(s)	Q1	Q2	Q3	Q4	Q5	Q6	Q7	Q8	Q9	Total Score	Decision to Include
McCullogh [40]	Yes	Yes	No	No	Yes	No	Yes	Yes	Yes	Moderate	Include
Singh [41]	Yes	Yes	No	No	Yes	No	Yes	Yes	Yes	Moderate	Include
Abiola et al. [42]	Yes	N/A	N/A	No	Yes	Yes	Yes	Yes	Yes	Moderate	Include

**Table A4:** Appraisal of Randomized Controlled Trial.

Author(s)	Q1	Q2	Q3	Q4	Q5	Q6	Q7	Q8	Q9	Q10	Q11	Q12	Q13	Total Score	Decision to Include
Haywood et al. [29]	Yes	No	Yes	No	No	No	Yes	Yes	Yes	Yes	Yes	Yes	Yes	High	Include
